# Project Optimus, an FDA initiative: Considerations for cancer drug development internationally, from an academic perspective

**DOI:** 10.3389/fonc.2023.1144056

**Published:** 2023-03-03

**Authors:** Ravindhi Murphy, Sarah Halford, Stefan Nicholas Symeonides

**Affiliations:** ^1^ Centre for Drug Development, Cancer Research UK, London, United Kingdom; ^2^ Edinburgh Experimental Cancer Medicine Centre, University of Edinburgh, Edinburgh, United Kingdom

**Keywords:** Project Optimus, FDA – Food and Drug Administration, cancer, oncology, drug development [MeSH], academic, international, United Kingdom

## Abstract

Modern cancer therapeutics are increasingly targeted, bringing the promise of new and improved activity, alongside better tolerability. However, while many are indeed resulting in dramatic improvements in disease control and patient survival, short- and long-term tolerability has not always accompanied it. The choice of dose and schedule is often in the upper range of the therapeutic window, driven by the maximum tolerated dose (MTD) model of previous cytotoxic agents. There is increasing recognition that this needs to change, by taking a more holistic approach to determine the optimal dose for desired biological effects and tolerability early in clinical development. In the US, the FDA’s Oncology Centre of Excellence is addressing this *via* the Project Optimus initiative: aiming to reform dose optimisation studies so that they can demonstrate the most appropriate dose selection. Early clinical development will need to demonstrate the dose-exposure, -pharmacodynamic, -toxicity and -activity relationships, including randomised evaluations for dose selection. Regulatory agencies outside the US are similarly exploring this. Along with Australia, Brazil, Canada, Israel, Singapore and Switzerland, the UK participates in Project Orbis, a collaborative program with the FDA to accelerate patient access to new cancer medicines through coordinated regulatory review. Close alignment with Project Optimus will be important internationally and will require changes across industry, including for academic units and small biotech. We discuss our perspective on the implications, and opportunities, for early phase oncology trials as a uniquely charity-funded drug development facility, the Centre for Drug Development within the Cancer Research UK charity.

## Context and international relevance

The landscape of clinical trial development in oncology has evolved from studies primarily evaluating chemotherapeutics, to those that are now evaluating an ever-widening range of novel therapeutics including molecularly targeted agents, biologics and immunotherapies, as single agents or in combination. Historically, the MTD has often been synonymous with the recommended phase 2 dose, with the assumption that the dose-activity relationship continues in line with the dose-toxicity relationship, producing meaningful increases in activity even at the limit of tolerability. This assumption was very relevant for conventional chemotherapy that affects all dividing cells in a dose-dependent manner and so formed the framework for early phase oncology clinical design for decades. However, current therapeutics, which generally target a more cancer-specific vulnerability, often have significantly less on-target toxicity and their activity-toxicity relationships are not as closely linked. The optimal dose may no longer be the MTD, requiring new approaches for early phase oncology trials.

In 2021, the FDA Oncology Centre of Excellence announced Project Optimus, an initiative to reform and improve dose optimisation and selection in oncology drug development (1–4), which will have implications on current and future drug development pipelines in the US and internationally.

Oncology is a key global therapy area for drug development and regulatory approval worldwide, and Project Optimus will have direct international reach. For instance, Project Orbis is a global collaborative program, also launched by the US FDA Oncology Centre of Excellence, which aims to expedite patient access to new cancer medicines in the USA and internationally through a framework of parallel regulatory submission and review (5, 6). This will naturally also bring closer international alignment with FDA initiatives, including Project Optimus. The seven global regulatory partners are Australia, Brazil, Canada, Israel, Singapore, Switzerland and the UK, and this has been particularly relevant for the UK after its departure from the European Union (EU) in 2020 necessitated significant healthcare reform and additional international collaborations.

Academic drug discovery is a vital component of drug development, collaborating with, and complementing, biotech and pharmaceutical industries (7). Academic discoveries drive new therapeutic opportunities and the in-depth biological knowledge and translational research expertise found within academic institutions is a vital resource to improve success rates in clinical development (8). The Cancer Research UK (CRUK) Centre for Drug Development (CDD) is the world’s only charity-funded drug development facility which partners with industry and academia in early phase cancer drug development. The CDD portfolio would be equivalent to that of a medium-sized pharmaceutical company and its unique funding and strategy provides a relevant viewpoint on some of the pertinent challenges in cancer drug development in both academic and biotech settings (9). In this article we discuss the implications, challenges and opportunities of Project Optimus for international drug development and highlight academic considerations.

## Problem being addressed

The FDA had previously noted trends of high rates of dose reductions and intolerability during new drug application review for targeted agents, suggesting inadequate characterisation of drugs prior to registration trials (4). There is additional real-world data that reflects high rates of dose reduction in even well-established targeted agents such as lenvatinib, regorafenib and everolimus (10–14). A review of the tolerability of molecularly-targeted agents in phase 3 trials at doses and schedules recommended by phase 1 trials identified that 48% of patients required dose modification (15). Toxicities may be acute, such as those seen in typical dose-finding studies, or they may be chronic low-grade toxicities that are often less characterised pre-registration. Exposure to both these acute and chronic toxicities raises questions of whether we are starting our drugs at the most appropriate dose level but it also raises questions about extrapolating efficacy data when the resultant dose modifications lead to lower dose intensities. It is therefore critical to have established whether these lower doses are still within a pharmacodynamically active range.

Dose escalation in early phase drug development has focused on determining the MTD, often with a traditional 3 + 3 design. Although more sophisticated models are being more widely used, such as CRM, BOIN and many others, improvements have generally been in the efficiency and reliability of determining that MTD (16, 17) and continue to provide limited data at other dose levels. This can inadequately characterise the true range and dose-dependency of exposure, pharmacodynamics, activity and toxicity, limiting the ability to define the minimum reproducibly active dose (MRAD) and to optimise dose selection (18). This is particularly relevant for current molecularly targeted agents, biologics and immunotherapies that often have target saturation limits below the MTD, suggesting that those intermediate dose levels may have similar efficacy with potentially fewer off-target side effects. Current agents are also typically administered continuously until disease progression (unlike the limited courses of many chemotherapies), and with more durable benefit, so long-term tolerability, including late toxicities and the management of persistent low-grade toxicity, are increasingly significant issues. Poorly characterised doses and schedules may therefore lead to unnecessarily high rates of acute and chronic toxicities, with consequent dose interruptions, reductions to under-characterised dose levels, and premature discontinuations. This may comprise current drug exposure, while persistent, non-reversible toxicities may also impact future lines of therapy.

## Trial requirements

Friends of Cancer Research is an organisation driving collaborations between healthcare partners to improve policy change, support ground-breaking science and expedite access of new cancer therapies. Their 2021 white paper on dose optimisation has set the scene for how to address this challenge, highlighting findings from stakeholders in industry, academia, the FDA and patient advocacy groups with recommendations to support adequate dose optimisation studies (19).

Dose-finding studies at the earliest stages of drug development will need to adequately characterise the dose-exposure, -pharmacodynamic, -toxicity and -activity relationships in order to select the most appropriate dose and schedule be brought into the registrational trial and subsequently used post approval (20, 21). There is an expectation for expansions at multiple dose levels to characterise the therapeutic window, for more extensive and highly validated pharmacodynamic analyses, and for granularity on tolerability, patient quality of life impact and late toxicities. Readouts of efficacy biomarkers in more selective, and potentially earlier-line, populations will be encouraged from the earliest stages of development and must be well-defined.

Project Optimus emphasises the need for more robust understanding of the impact of different doses on efficacy and toxicity and randomised dose comparisons are expected to be necessary early in development. Whilst randomised comparison of at least two doses is increasingly important, this does not need to be powered for rigorous statistical comparison, rather it needs to be sufficiently sized to understand the general shape of the dose relationship. As a minimum, comparison of the minimal biologically active dose (likely estimated from pharmacokinetic-pharmacodynamic (PK-PD) modelling) to the highest tolerable dose will be required, to explore if there is a clear differential benefit with acceptable tolerability. Such doses would need to be selected without overlapping PK exposures (i.e., 2-3 fold apart). More limited data from intermediate doses may be integrated to understand the general shape of the dose relationship and guide dose selection.

## Issues and implications

Whilst using the minimal biologically active dose seems appealing, there are potential concerns with this approach that must be considered and addressed.

### Intrapatient titration

For some therapeutics, starting at an initial high dose, then tapering to tolerability can be a strategy to drive response e.g. of the various anti-PD-1/L1 plus VEGFR inhibitor combinations in renal cell carcinoma, lenvatinib plus pembrolizumab involves a relatively high initial dose of the VEGFR inhibitor (20mg lenvatinib) and has the highest reported response rate, even if subsequent dose reduction are common and survival benefit is very similar to the other combination (22). In contrast, a reduced dose of the VEGFR inhibitor cabozantinib (23, 24) was given in its combination with nivolumab, which prioritises tolerability and may have supported the positive quality-of-life endpoints for this combination. Again, survival benefit was similar to other combinations. That choice between prioritising response or tolerability will be patient-dependent but at least the Project Optimus framework allows exploration of both dosing strategies to inform those decisions. It may also lead to increasing intermediate dosing options, as the 40mg dose of cabozantinib now used only has a 20mg dose as the next approved dose reduction.

### Individualised risk: benefit assessment

Even small incremental increases in activity at doses that begin to push tolerability may be considered worthwhile by some patients, especially if there are improvements in long-term outcomes (as in early disease or, increasingly, in sensitive subpopulations with advanced disease). An example of this is the addition of ipilimumab to nivolumab in metastatic melanoma, where nivolumab alone may be only marginally less effective but significantly better-tolerated (25). The optimal dosing and schedule of this combination continues to be refined post-registration and extended dosing intervals were not explored pre-registration but are now demonstrating increased tolerability without compromising efficacy (26).

### Heterogeneity

Minimal biologically active dose may not always be consistent across different populations, including tumour type (due to additional pathways and feedback signalling); specific mutations (that may differ for drug affinity or inhibitory activity), and even disease stage (due to physiological interactions or the importance of control versus clearance), perhaps especially for multi-kinases inhibitors. There may well also be reduced affinity for resistance and partial-resistance mutations, which may reduce durable control for doses that have similar initial activity. Although, arguably, Project Optimus builds the framework data to explore tailored schedules.

### Complexity

Trial designs will need to evolve. It may be necessary to increase intervals between dose cohorts to capture PD and longer-term toxicities and/or a shift to model-based systems that can integrate later data, e.g. TiTE-CRM (27, 28), but may have increased resource requirements. Dose-finding models will need to deal with the associated complexity of integrating data on broad tolerability (not just DLTs) into dose-finding models, alongside PD and activity measures. The timing and prioritisation of dose comparisons will need to be reconciled with other expansion priorities such as population optimisation (refining tumour types and selection biomarkers) which may be needed before dose-efficacy relationships can be explored in an adequately sensitive population. It is recognised that emphasis will be placed on strategies such as using real-time PK data and early validation of PD biomarkers to guide development.

### Pace

The pace of drug development may be affected by the need for increasingly rigorous dose characterisation. Whilst on average this may be balanced by yielding more optimal dosing for registration studies, it may especially impact fast track agents, such as sotorasib, that have undergone further dose comparisons where they might previously have proceeded straight to registration (21). Although this may be built into future early phase designs, as these highly active fast track agents generally validate activity early, giving confidence to fund multiple large dose expansions, with interpretable levels of radiological responses – e.g. osimertinib (29) and others. Additionally, streamlined regulatory interactions such as Fast Track in the USA and ILAP in the UK may help overcome the challenge of increased timelines.

### Cost

Changes associated with Project Optimus may increase the initial cost of drug development, with implications on long term affordability of new drugs (30, 31). While larger pharmaceutical companies may be able to rapidly expand budgets to include additional, randomised cohorts in response to early promising signals, academic centres and biotech often have less flexibility. This will necessitate smarter trial designs that make most efficient use of participant numbers, including integrating comprehensive drug characterisation and dose comparisons throughout, and amplifying the available data *via* intra-patient comparisons of PK-PD (which we are now increasingly using). It is likely that changes in trial design, data requirements and costs will have implications for inflection points and triggers for next funding series in biotechs.

### Biomarker validation

The need for validated biomarkers to enable optimal patient selection and PK-PD decision-making from the earliest stages will require earlier and more extensive biomarker development. This brings implications in terms of resource requirements and cost, although academic institutions may be able to leverage in-house and collaborative biomarker expertise.

### Interpretable response endpoints

Differentiating doses will be more challenging where responses rates are lower, or interpretation affected by combinations. While PD endpoints will add to this data, appropriate and accessible PD measures will depend on the mode of action and may require increased tumour biopsies. However, this can be mitigated by leveraging advances in radiographic, ctDNA and blood biomarkers, allowing multiple timepoints, less variability, and great accessibility and acceptability than biopsies.

### Statistical power

Exploring multiple factors with the small numbers seen in early phase trials can compromise statistical rigor. Confirmatory cohorts or Bayesian approaches may help.

### Interim analyses

Interpreting mid- to long-term outcome measures can be compromised when there are cohort, or individual patient, treatment crossovers or dose modifications based on interim analyses. However, these do provide an opportunity to explore intra-patient dose-relationships.

## Opportunities

The above issues are surmountable, and Project Optimus brings new opportunities to cancer drug development, including for academic units.

### Delivering more patient-centred drug development

This is a key opportunity for quality of life measures to be taken into consideration earlier in development. Increased attention on patient involvement is encouraged and the FDA has set out methodological patient-focused drug development (PFDD) guidance documents to help stakeholders collect and use robust and meaningful patient input (32). Academic and charity-funded institutions such as CDD have close links to patient groups, allowing their views and input to help shape clinical trial development. Patients are uniquely placed to contribute to the understanding of benefit and risk considerations during drug development with a range of opinions and wishes. The use of patient reported outcome measures (PROMs) can help define true tolerability behind CTCAE gradings, particularly in relation to chronic low-grade toxicities.

### Building models to optimise and adapt dosing schedules

Better characterisation of dose-relationships is an opportunity to explore inter-patient variability and the personalisation of dosing. Improvements in population-PK modelling may build more robust models against which future schedule changes or flat dosing can be assessed. It may allow exploration of the duration of therapy and its contribution to chronic toxicities, and the role of intermittent schedules [e.g. sunitinib (33)], or adaptive dose titrations [e.g. axitinib (34)], in managing cumulative toxicities. Dose optimisation platforms may allow earlier exploration of drug-drug interaction, food and diurnal effects. Finally, early understanding of dose-relationships may enable combination therapies, new schedules and re-formulation improvements earlier in development, which may expedite approvals at a later date.

### Producing clinical data that better represents real-world populations

A concerted effort is required across stakeholders to increase equity, diversity and inclusion in cancer trial participation (35). A key feature of Project Optimus is an emphasis on representing patients from a diverse range of settings (36). Academic and charity-funded institutions are well-placed to connect with under-represented populations such as ethnic minorities and socioeconomically disadvantaged groups. Public health systems, such as the UK’s National Health System, are well placed to recruit patients from both socioeconomically and ethnically diverse backgrounds, which is paramount for ongoing clinical trials.

### Scientific innovation and collaboration in novel models for drug development

Academic cancer drug development centres may be uniquely advantaged to enact these changes (37, 38). They often have significant statistical expertise, including designing and implementing novel trial designs (e.g. future dose-finding models that can include randomisation and cohort expansions to integrate the exploration of dose-dependency). Also, translational science expertise in academic centres can aid more representative preclinical models, more accurate PK-PD modelling, a deeper understanding of biology and biomarkers, advances in translational assays including surrogate tissue, and the use of big data (e.g. in identifying biomarkers, subpopulations and associations). Clinical expertise in the associated trials units can complete the iterative loop of identifying and reacting to emerging trial data, while also linking in with the patient voice and experience across diverse populations. Finally, they can leverage a network of academic collaborators, bringing together complimentary expertise and innovations, and also expanding capacity, including access for patients to these innovative new trials and treatments.

## Conclusions

Optimising dose selection in oncology is crucial to improve durable clinical outcomes for patients and enable meaningful decisions on risk: benefit balance, particularly in this exciting age of increasingly targeted anticancer agents. Previous methodologies do not adequately characterise the essential dose-exposure-toxicity-response relationships (4). The principal goal of Project Optimus is to support a paradigm shift from using MTD as the default approach for oncology drug development to a randomised exploration of optimal dosing. It is envisioned that more rigorous selection of dose schedules will help patients gain increased benefit from systemic cancer treatment, with reduction of debilitating or chronic side effects. While there are some challenges to be considered, academic drug development units are well-placed to rise to those challenges through innovative trial designs, with novel statistical methodologies, representative models, clinical expertise and the input of our patients, to deliver optimised, patient-focused drug development.

## Data availability statement

The original contributions presented in the study are included in the article/supplementary material. Further inquiries can be directed to the corresponding authors.

## Author contributions

RM, SH, and SNS contributed to conception and planning of the manuscript, coordinated by SNS. RM wrote the first draft of the manuscript. RM, SH, and SNS wrote sections of the manuscript. All authors contributed to the article and approved the submitted version.
